# Emotional regulation and body dissatisfaction: the mediating role of anger in young adult women

**DOI:** 10.3389/fpsyt.2023.1221513

**Published:** 2023-07-17

**Authors:** Janire Momeñe, Ana Estévez, Marta Herrero, Mark D. Griffiths, Leticia Olave, Itziar Iruarrizaga

**Affiliations:** ^1^Psychology Department, School of Health Sciences, University of Deusto, Bilbao, Spain; ^2^International Gaming Research Unit, Psychology Department, Nottingham Trent University, Nottingham, United Kingdom; ^3^Faculty of Health Sciences, Universidad Internacional de Valencia, Valencia, Spain; ^4^Department of Experimental Psychology, Cognitive Processes and Speech Therapy, Faculty of Social Work, Complutense University of Madrid, Madrid, Spain

**Keywords:** body dissatisfaction, emotion regulation, anger, inhibition of emotional impulses, young adult women, path analysis

## Abstract

**Introduction:**

Emotion regulation difficulties have an important role in the presence of negative self-image. These problems in the self-regulation of emotion could lead to negative emotional processes (such as anger) that can lead to body dissatisfaction. Therefore, the aim of the present study was to examine emotion regulation difficulties than can negatively impact self-image and to understand if anger acts as mediator in the relationship between emotion regulation and body dissatisfaction.

**Methods:**

A cross-sectional study was carried out comprising 565 young adult women aged 18–30 years. The participants were administered the Difficulties in Emotion Regulation Scale (DERS), the State-Trait Anger Expression Inventory 2 (STAXI-2), and the Body Dissatisfaction dimension of the Eating Disorder Inventory-2 (EDI-2). The proposed hypotheses were tested by path analysis in MPlus 8.0.

**Results:**

The results indicated that anger had a positive significant effect on body dissatisfaction as well as the non-acceptance of emotional responses, the lack of emotional awareness, and the lack of emotional clarity. Of all the dimensions of emotional regulation difficulties, impulse control difficulty was the dimension which had a positive significant indirect effect on body dissatisfaction explained by increased anger.

**Discussion:**

The present study suggests the importance of emotion regulation in the prevention of body dissatisfaction. Impulse control difficulty may be the key emotion regulation emotion in explaining the increments of anger that lead to body dissatisfaction. Among young adults, the promotion of positive body image can be promoted by helping this population to self-regulate their anger impulses.

## Introduction

Body dissatisfaction is defined as an individual’s negative evaluation of his or her own body due to the discrepancy between the perceived actual body shape and the desired ideal (1). In recent years, there has been an increase in body dissatisfaction predominantly among young adult women due to different risk factors such as (i) appearance pressures, (ii) internalization and comparisons, and (iii) need to conform to societally established beauty ideals (2). This has been associated with many negative health consequences, both physical and psychological, resulting in a negative impact on self-esteem, mood, social functioning (3) and overall health (4).

Body dissatisfaction is prevalent in the general population, but it is also a potential risk factor for the development of eating disorders (EDs) (5) and represents a central psychopathological feature of this issue (6). Therefore, the need for further research has been called for to delineate the factors associated with body dissatisfaction with the aim of improving treatment efficacy due to its increasing prevalence worldwide and its impact on physical and mental health (6, 7).

In recent years, self-discrepancy theory (8) has been used to help explain body dissatisfaction. This theory posits that the current or actual self, that is how individuals perceive themselves, is compared with the future self (how individuals wish to become) and with the should self (the attributes that should be possessed related to sociocultural expectations). It has been shown that this discrepancy between actual and ideal body image is related to higher levels of negative emotions such as depression and anxiety and difficulties in emotion regulation (9, 10). Therefore, negative emotional states and difficulties in emotion regulation are risk factors for the development and increase in body dissatisfaction (11, 12).

Additionally, difficulties in emotional regulation, that is, difficulties by individuals in influencing the onset, intensity, and duration of their emotions (13) predict the onset, maintenance, and severity of dysfunctional eating behaviors (14, 15). Related to this, recent studies have shown that individuals who employ adaptive emotional regulation strategies report lower body dissatisfaction (12, 16). The ability of individuals to be able to flexibly regulate their emotions is critical for adaptive functioning across the lifespan because it is an important factor that impacts overall psychological wellbeing (17).

Regarding the influence of negative emotional states on the development of body dissatisfaction, this is consistent with Alireza and Padhy (1), who observed that body dissatisfaction toward the body in general (or toward specific body parts) changes with mood. Previous studies have focused on the study of depression and anxiety (18). However, anger understood as an emotional reaction to threat, frustration, or social provocation, and which modulates an individual’s risk of aggression could also play an important role in the development of body dissatisfaction because previous literature has associated anger with the increase and prediction of eating disorder symptoms (19–23).

In addition, individuals with EDs tend to repress and internalize their anger excessively (24, 25). This is because individuals with EDs perceive the expression of anger as potentially negative and “dangerous” because it has the potential to cause rejection (26). Consequently, individuals direct anger at themselves through ED symptomatology. Related to this, individuals’ expression of anger toward themselves in the form of non-suicidal self-injury and self-injury attempts are frequent among those with EDs and are indicative of increased eating pathology (27, 28). Therefore, inhibited expression of negative emotions is related to cognitive and affective components of body image dissatisfaction (21, 29).

As demonstrated, difficulties in emotional regulation and negative emotions play an important role in the development and maintenance of EDs. Although the predictive role of difficulties in emotional regulation among individuals with EDs is well established, the relationship between difficulties in emotional regulation and body dissatisfaction has been little studied, as well as the factors that may influence the association. Likewise, although the increase in body dissatisfaction in the face of negative emotions such as depression, anxiety, and stress has been investigated (18), few studies have explored anger as a psychological factor associated with body dissatisfaction among young women despite being a particularly important trait in the development of EDs.

In addition, it has recently been noted that current treatments for EDs have limited long-term success, in part because the psychological processes involved are not well understood (30). Moreover, the need to study other negative emotional states that may be related to EDs has been noted (31). To address this gap, the present study examined how difficulties in emotional regulation are related to body dissatisfaction through anger among young adult women. Given this, it was hypothesized that: (i) as difficulties in emotional regulation increase, so will anger and body dissatisfaction in parallel; (ii) the greater the anger, the greater the body dissatisfaction; and (iii) anger could play an important role in the relationship between difficulties in emotional regulation and body dissatisfaction. Based on self-discrepancy theory, a greater discrepancy between the real self and ideal self may generate higher levels of anger among individuals who have difficulties in emotional regulation which could subsequently lead to greater body dissatisfaction.

## Materials and methods

### Participants

A total of 565 young women aged 18–30 years (*M* = 20.72 years, *SD* = 2.29) participated in the study. As shown in Table 1, these young women were mostly students, heterosexual, and unmarried. Almost 40% reported to have been in psychological therapy at least once in their lifetime but less than a 5% were taking psychiatric medication at the time of the study. Of those who had been in therapy, the main motivations for treatment were depressive disorders, family problems, anxiety disorders, and eating disorders.

**TABLE 1:** Sociodemographic characteristics of the study sample (*n* = 565).

Variable	*N*	%
**Occupational status**		
Student	483	85.5
Worker	81	14.3
Other	1	0.2
**Civil status**		
Single	549	97.2
Married	1	0.2
Other	15	2.7
**Sexual orientation**		
Heterosexual	464	82.1
Bisexual	76	13.5
Homosexual	25	4.4
Attended therapy in life*		
Yes	214	39.3
No	331	60.7
Psychiatric medication**		
Yes	19	3.36
No	546	96.64
Reason for therapy attendance***		
Depressive disorder	51	23.94
Family problems	48	22.54
Anxiety disorder	46	21.60
Eating disorder	16	7.51
Behavioral disorder	9	4.23
Bullying	8	3.76
Neurodevelopmental disorder	7	3.29
Self-esteem	6	2.82
Gender violence	6	2.82
Trauma/stressor related disorders	3	1.41
Adaptative problems	3	1.41
Couple problems	2	0.94
Interpersonal difficulties	2	0.94
Psychotic disorder	1	0.47
Dissociative disorder	1	0.47
Personality disorder	1	0.47
Elimination disorder	1	0.47
Sexual dysfunction	1	0.47
Obsessive-compulsive disorder	1	0.47

*This variable contains missing data; **This variable was answered only for those who took medication; ***This variable was answered only for those who answered positively to attending psychological therapy.

### Measures

#### Body dissatisfaction

The “body dissatisfaction” subscale of the Eating Disorders Inventory-2 [EDI-2; (32); Spanish version: (33)] was used to assess body dissatisfaction. The EDI-2 assesses the symptoms that accompany EDs such as anorexia nervosa and bulimia nervosa. It comprises 91 items across 11 subscales that evaluate central aspects of EDs. Three subscales assess attitudes and behaviors related to food and weight and the other eight subscales assess clinically relevant psychological traits in these types of disorder. The body dissatisfaction subscale assesses dissatisfaction with overall body shape (e.g., “*I think my thighs are too thick*”, “*I think my hips are too wide*”). Items are rated using a Likert-type format from 0 (*Never*) to 5 (*Always*) and summed to provide a total score on the subscale. In the present study, the Cronbach’s alpha coefficient for the body satisfaction subscale was 0.89.

#### Emotion regulation difficulties

The Difficulties in Emotion Regulation Scale [(34); Spanish version: (35)] was used to assess emotion regulation difficulties. The scale comprises 28 items assessing five emotional regulation deficits. “Impulse control difficulties” assesses individuals’ feeling of overflow due to emotional intensity and the feeling of persistence of negative emotional states (e.g., “*When I feel bad, I lose control*”, “*When I feel bad, I think I will be like this for a long time*”); “difficulty engaging in goal-directed behavior” assesses individuals’ difficulties concentrating or performing tasks when experiencing negative emotions (e.g., “*When I feel bad, I have difficulty concentrating*”); “lack of emotional awareness” assesses individuals’ difficulties attending to and admitting to their own emotional states (e.g., “*I am attentive to my feelings*”); “lack of emotional clarity” assesses individuals’ difficulty in knowing and understanding their emotions (e.g., “*I am confused about what I feel*”); “non-acceptance of emotional responses” assesses individuals’ tendency to experience secondary negative emotions in response to a primary negative emotion (e.g., “*When I feel bad, I feel ashamed of myself for feeling that way*”). Items are rated using a Likert-type format from 1 (*Almost never*) to 5 (*Almost always*). A higher score obtained on each subscale, as well as in the total scale, suggests that the person shows greater difficulties in emotional regulation. In the present study, good to excellent internal consistencies were obtained for impulse control difficulties (α = 0.90); non-acceptance of emotional responses (α = 0.91); difficulty engaging in goal-directed behavior (α = 0.89); lack of emotional awareness (α = 0.74); and lack of emotional clarity (α = 0.79).

#### Anger

The “general index of anger expression” in the State-Trait Anger Expression Inventory [STAXI-2; (36); Spanish version: (37)] was used to assess anger in the present study. The STAXI-2 comprises 49 items across six scales and five subscales and provides a general test index. As aforementioned, only the “general index of anger expression” was used (e.g., “*I get angry easily*”, “*I express my anger*”). The Cronbach’s alpha in the present study was 0.85.

### Procedure

The study design was cross-sectional. Participants were recruited in two ways: online and face-to-face. Participants recruited online accessed the study through an online platform^1^ which contained the study survey. Participation was promoted through different social networks (e.g., *Twitter, LinkedIn, Instagram, Facebook*) as well as advertisements on research websites. The participants recruited face-to-face answered the survey in “pencil and paper” format at localities accessible to the research team (e.g., university campus, gyms). The only exclusion criterion was being under 18 years of age. All participants provided informed consent to participate in the study. Online participants clicked a button indicating that they had read the information and agreed to participate voluntarily in the study. Face-to-face participants ticked the corresponding box on the paper. Likewise, participants were informed that they could leave the study at any time. The study obtained the ethical approval for the study was provided by the Deontological Commission of the Faculty of Psychology of the first author’s university (Ref. 2020/21-035).

### Data analysis

First, the descriptive statistics were computed and preliminary analyses were performed to test the relationship in between the sociodemographic variables and the dependent variables (i.e., anger and body dissatisfaction) with SPSS 18.0 (38). For the latter, student *t*-tests for independent samples and one-factor ANOVAs were applied for binary and multi-categorial variables, respectively. In addition, the normality assumption was tested. Based on Cain et al.’s (39) indications, normality is assumed when the asymmetry and kurtosis values are lower than |2|. As displayed in Table 2, the asymmetry and kurtosis of all study variables were in between −2 and +2 so normality was assumed. Additionally, and given the presence of normality, bivariate Pearson correlations were computed to examine the relationships between each pair of the study variables.

**TABLE 2:** Differences in the dependent variables by sociodemographic groups.

		Anger			Body dissatisfaction		
Variable	*n*	M (SD)	*t*	*p*	M (SD)	*t*	*p*
Occupational status			−2.36*	0.019		−0.09	0.928
Student	483	32.16 (10.65)			18.85 (10.92)		
Worker or other	82	29.16 (10.75)			18.73 (11.32)		
Civil status			−0.42			−0.22	
Single	549	31.75 (10.68)			18.85 (10.97)		
Married or other	16	30.63 (12.02)			18.25 (11.21)		
Attended therapy in life			1.25	0.213		1.68	0.094
Yes	214	32.64 (10.93)			19.86 (11.49)		
No	331	31.48 (10.43)			18.25 (10.51)		
Psychiatric medication			0.01			2.12*	0.035
Yes	94	31.96 (11.31)			21.09 (11.32)		
No	452	31.94 (10.49)			18.47 (10.81)		
Therapy for ED			−0.27	0.787		−1.36	0.175
Yes	16	32.44 (12.41)			22.50 (10.40)		
No	549	31.70 (10.66)			18.73 (10.97)		
**Variable**	***n***	**M (SD)**	***t***	***p***	**M (SD)**	***t***	***p***
Sexual orientation			1.86	0.157		1.88	0.153
Heterosexual	464	31.97 (10.59)			18.43 (10.78)		
Homosexual	25	33.36 (12.24)			19.72 (11.62)		
Bisexual	76	29.64 (10.76)			21.00 (11.76)		

*p < 0.05.

Second, the hypothesized model was tested with path analysis with Mplus 7.0 (40) to examine the dimensions of emotion regulation difficulties with higher relationship with anger and body dissatisfaction and the proposed mediation. The model was estimated with maximum likelihood because all the variables followed a normal distribution and there were no missing data (41). The five emotion regulation difficulties (i.e., impulse control difficulties, non-acceptance of emotional responses, difficulty engaging in goal-directed behavior, lack of emotional awareness, and lack of emotional clarity) were modeled as independent variables, anger as the mediator, and body dissatisfaction as the final dependent variable. In this model, the sociodemographic indicators with a significant relationship with any of the dependent variables were included as covariates to control for confounding effects.

The test the fit between the hypothesized model and the observed data, the chi-square test (χ^2^), the comparative fit index (CFI), the Tucker-Lewis index (TLI), the root mean squared error of approximation (RMSEA), and the standardized root mean square residual (SRMR) were computed. CFI and TLI values around or over 0.95, RMSEA around or lower 0.06, and SRMR values around or lower than 0.08 were considered indicators of good fit (42). In addition, *r*^2^ was examined as effect size of the model so *r*^2^ > 0.01 were considered indicators of small effect size, *r*^2^ > 0.09 medium effect sizes and *r*^2^ > 0.25 large effect sizes (43).

Because the distribution of the indirect effects does not follow normality and can lead to computation bias (44), the estimation of the indirect effects of the emotion regulation difficulties on body dissatisfaction mediated by anger were tested following Stride et al.’s (45) coding examples with 10,000 bootstrap samples to reduce these biases.

Preacher and Coffman’s (46) web software was used to compute the statistical power of the proposed covariance structure model using RMSEA. For an alpha of 0.05, a sample size of 565, a model with three degrees of freedom, a null RMSEA of 0.00, and an alternative RMSEA of 0.08, the associated analytical power was 0.80 which indicates an adequate statistical power to test the model.

## Results

In Table 1, the sociodemographic characteristics of the sample are described, and their relationships with the dependent variables are shown in Table 2. These analyses indicated that (i) being a student was significantly related to higher anger and (ii) taking psychiatric medication was significantly related to higher body dissatisfaction. Moreover, age showed a significant correlation with body dissatisfaction (*r* = −0.14, *p* = 0.001), but not with anger (*r* = −0.08, *p* = 0.062). No other relationship with the sociodemographic variables was significant.

As shown in Table 3, all study variables were positively and significantly correlated. Therefore, higher levels of all the indicators of emotion regulation difficulties were related to higher anger and body dissatisfaction. Moreover, higher anger was related to higher body dissatisfaction.

**TABLE 3:** Descriptive statistics and bivariate correlations of the study variables.

	Descriptive statistics				Correlations					
Variable	*M*	SD	As	Kr	1	2	3	4	5	6
**Emotion regulation difficulties**										
1. Impulse control difficulties	17.34	7.44	1.08	0.57						
2. Non-acceptance of emotional responses	15.09	7.01	0.86	−0.09	0.67***					
3. Difficulty engaging in goal-directed behavior	10.83	4.13	0.42	−0.70	0.68***	0.52***				
4. Lack of emotional awareness	9.75	3.24	0.30	−0.44	0.19***	0.26***	0.12**			
5. Lack of emotional clarity	9.35	3.23	0.78	0.30	0.51***	0.50***	0.41***	0.49***		
6. Anger	31.72	10.71	0.04	−0.54	0.50***	0.31***	0.37***	0.23***	0.31***	
7. Body dissatisfaction	18.83	10.97	0.26	−0.80	0.32***	0.40***	0.29***	0.28***	0.33***	0.23***

As = Asymmetry; Kr = Kurtosis. **p < 0.01; ***p < 0.001.

**FIGURE 1:**
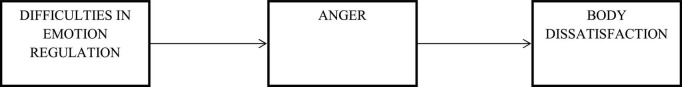
Hypothesized model.

The hypothesized model was tested with path analysis including the sociodemographic indicators with significant effects on the dependent variables as covariates (see Figure 1) (i.e., being student as covariate of anger, and age and taking psychiatric medication as covariates of body dissatisfaction). The model fit indicators outdid the cut-off standards [χ^2^(3) = 2.98, *p* = 0.395; CFI = 1.00; TLI = 1.00; RMSEA < 0.01; SMRM = 0.01] which indicated that the hypothesized model fitted the data adequately. This model explained 30% of the variance in anger and 19% of the variance in body dissatisfaction which indicated a large and medium effect size, respectively.

**FIGURE 2:**
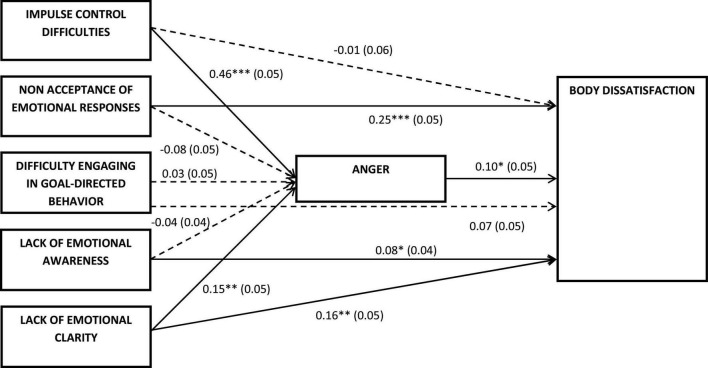
Standardized path coefficients and standard errors of the direct effects of the model. Dash lines represent non-significant direct effects. *p < 0.05, ^**^p < 0.01, ^***^p < 0.001.

The direct effects shown in Figure 2 indicate that impulse control difficulties and lack of emotional awareness had significant effects on anger, but not on the other emotion regulation difficulties. Therefore, greater difficulties with impulse control were related to greater anger while the lack of emotional awareness was related to lower anger. Regarding body dissatisfaction, the direct effect of anger on body dissatisfaction was significant. Therefore, higher levels of anger were associated with greater body dissatisfaction. In addition, non-acceptance of emotional states and lack of emotional clarity also had a significant effect on body dissatisfaction. Therefore, higher levels of these emotion regulation difficulties were associated with higher body dissatisfaction. Impulse control difficulties, difficulty engaging in goal-directed behavior, and the lack of emotional awareness were not directly associated with anger.

The indirect effects of the emotion regulation difficulties on body dissatisfaction were also tested (see Table 4). Of the five emotion regulation difficulties, only impulse control difficulties had a significant indirect effect on body dissatisfaction. Therefore, higher impulse control difficulties were associated with higher body dissatisfaction through the effect on anger.

**TABLE 4:** Standardized indirect effects of emotion regulation difficulties on body dissatisfaction through anger.

Indirect effect	Effect	SE	95% CI
Impulse control difficulties → Anger → Body dissatisfaction	0.054*	0.025	[0.006, 0.103]
Non-acceptance of emotional responses → Anger → Body dissatisfaction	-0.008	0.006	[−0.020, 0.004]
Difficulty engaging in goal-directed behavior → Anger → Body dissatisfaction	0.006	0.006	[−0.006, 0.017]
Lack of emotional awareness → Anger → Body dissatisfaction	-0.014	0.008	[−0.029, 0.001]
Lack of emotional clarity → Anger → Body dissatisfaction	-0.007	0.005	[−0.017, 0.003]

SE = Bootstrapped standard error; 95% CI = Bootstrapped 95% confidence interval. *p < 0.05.

## Discussion

The first objective of the present study was to analyze the relationship between difficulties in emotional regulation, anger, and body dissatisfaction. The results showed that all the variables of the study were positively and significantly correlated with each other. Therefore, higher levels of difficulties in emotion regulation were related to greater body dissatisfaction. There is a consensus in previous literature that difficulties in emotion regulation play an important role in the etiology, maintenance, and severity of EDs. Accordingly, previous studies have shown that as difficulties in emotion processing and regulation increase, the symptomatology of EDs also increase in parallel (13–15, 30, 47–49). However, studies investigating the relationship between difficulties in emotional regulation and body dissatisfaction have been scarce, although in recent years they have become more prevalent.

Shriver et al. (16), recently noted that the use of adaptive emotional regulation strategies such as acceptance and reappraisal are associated with lower body dissatisfaction (12). For their part, McComb and Mills (50) suggested that difficulties in emotional regulation based on rumination and catastrophizing exerted an important role in body dissatisfaction. These results were similar to Rivière et al. (51), who noted that rumination was an important factor in the development and maintenance of body dissatisfaction. In line with these results, Lev-Ari et al. (52) reported that difficulties in emotional regulation were predictors of body dissatisfaction.

The results of the present study also suggested that higher levels of emotion regulation difficulties were related to greater anger. These results are consistent with those reported in previous studies where feelings of anger have been found to increase as difficulties in emotion regulation increase (19, 20), especially those related to suppression or rumination (17).

The present study also found that greater anger was associated with greater body dissatisfaction. Although this relationship has been scarcely examined in previous studies, these results can be explained by studies associating negative emotions such as depression, anxiety, and stress with increased body dissatisfaction (11, 12). Moreover, body dissatisfaction changes with mood. That is, in the presence of positive mood states, body dissatisfaction decreases, whereas in the presence of negative mood states body dissatisfaction intensifies (1).

Similarly, previous studies have associated anger with eating disorders. Greater symptoms of anorexia nervosa and bulimia nervosa have been reported among individuals with greater inappropriate anger (20, 22, 53). Furthermore, it has been found that following anger induction among patients with EDs, there was a significant increase in body size estimation (54). It has also been noted that individuals with an ED report higher levels of anger and a tendency to internalize and repress it compared to individuals without this problem (55). In line with this, inhibited expression of negative emotions is related to cognitive and affective components of body image dissatisfaction (1).

Secondly, the hypothesized model in which anger acted as a mediator in the relationship between difficulties in emotional regulation and body dissatisfaction was investigated. The results obtained showed that difficulties in impulse control and lack of emotional awareness had significant effects on anger. Likewise, anger had a significant positive effect on body dissatisfaction, where higher levels of anger were associated with greater body dissatisfaction. In turn, non-acceptance of emotional responses and lack of emotional clarity had a significant effect on body dissatisfaction, where higher levels of these emotional regulation difficulties were related to greater body dissatisfaction.

Among the emotional regulation difficulties, impulse control difficulties had a positive and significant indirect effect on body dissatisfaction, explained by increases in anger. Therefore, greater impulse control difficulties were related to greater body dissatisfaction through the effect on anger. These results can be explained by the fact that females with an ED tend to present a high need for control, and many authors have conceptualized eating disorders as pathologies of control (56, 57). This control is experienced as a source of pleasure and rebellion and control is required in both the external world and the internal world. Therefore, loss of control or, in other words, difficulties in impulse control produce an increase in discomfort and negative emotions. In turn, negative emotions or negative affect have been shown to increase body dissatisfaction (1). Furthermore, these results can also be explained by the fact that difficulties in emotional regulation influence the change in self-image (58). Likewise, specific negative emotional states such as anxiety and depression mediate the relationship between emotional regulation difficulties and dysfunctional eating behaviors such as emotional eating (59).

On the other hand, in relation to self-discrepancy theory (8), a recent study found that a decrease in difficulties in emotion regulation predicted a reduction in impulsive behaviors (such as binge eating), anxiety, and depression symptoms, and the discrepancy between the real and ideal self-image (60). Because of this, the results obtained suggest that greater difficulty in controlling impulses may generate higher levels of anger which, in turn, could influence the increase in the discrepancy between the real self and the ideal self and subsequently generate greater body dissatisfaction.

Moreover, affective regulation theory proposes that loss of control in eating is preceded by an increase in negative emotional states (61). Related to this, Braden et al. (62), noted that eating in response to anger was closely related to emotional regulation difficulties. In addition, the present study’s results can also be explained by the fact that reduced ability to control emotions and impulses and the tendency to suppress and deny negative emotional states have been associated with EDs (63, 64). Walenda et al. (31) also noted that individuals with eating disorders more frequently employ non-adaptive emotion regulation strategies such as rumination and suppression of negative feelings compared to non-eating disordered individuals. In turn, they noted that increased negative emotions predicted dysfunctional eating behaviors.

### Therapeutic implications

For their part, therapists treating patients with eating disorders should be aware of these relationships and consider the possibility that anger and impulse control difficulties may underlie and be an important focus in reducing body dissatisfaction among young women. Moreover, it is known that eating disorders have a function. That is, if something exists and persists over time, it must necessarily play an important role and have a positive utility. Otherwise, it would be destined to disappear (65). Therefore, by guiding individuals in the search for the positive and protective utility of their problem, they fight it less which can lead to its improvement. It is important to help females to explore whether they have difficulties in expressing anger, since in many of them a high need for complacency is present.

Consequently, the development of an ED is a non-verbal way of expressing distress and asking for help. Young women may be overwhelmed by the intensity of the emotions of anger and difficulties in impulse control and this can lead to body dissatisfaction as a way of narrowing the focus on what they can really control and gives them security which is body modification and obsession. Family-based therapy is one of the therapies of choice in EDs that attempts to address the entire family in order to construct explanatory hypotheses for the family distress and triggered symptomatology.

### Limitations and future research

The present study is not without limitations. First, the cross-sectional nature of the study precludes the analysis of causal relationships between variables. Future studies should replicate the study using longitudinal designs in order to provide a more robust view of the causal relationships between variables. A second limitation is the use of self-reports to collect the study data, as well as the use of subscales of psychometric instruments rather than the full scales. This is because there is a greater likelihood of recall bias in the study using self-reported data. Because of this, the use of explicit self-report measures could be limiting. In future, it would be useful to couple these measures to implicit/objective tasks as suggested by Scarpina et al. (66). Third, the study sample mainly comprised university students, which may limit the generalizability of the findings to other populations. Future studies could include and analyze females with different educational levels and from different cohorts. In addition, there could be other sociocultural factors that may have influenced and explained the relationship between the study variables such as sexual orientation, socioeconomic status or family dynamics.

When elaborating explanatory hypotheses, future studies could explore the possible presence of feelings of anger and their difficulty to externalize it, as well as to control impulses. This is due to the fact that it is common for females with an ED to have difficulties in differentiating themselves and achieving autonomy in their family environment, which can generate feelings of anger that cannot be expressed (67). In addition, dialectical behavioral therapy that works on emotional regulation should place special emphasis on the functional management of anger and impulse control (68).

Future studies should also aim to extend the present investigation and explore other variables involved in the relationship between difficulties in emotional regulation and body dissatisfaction. For example, future studies could investigate the mediating role of the use of social networking platforms in the relationship between difficulties in emotional regulation and the development of body dissatisfaction. Difficulty in impulse control plays an important role in the use of social networking platforms that may trigger greater body dissatisfaction. It would also be interesting to collect data on the BMI of the participants and analyze its influence on the study variables.

## Conclusion

The results of the present study suggest that difficulties in impulse control are a key factor in explaining the increased anger leading to body dissatisfaction among young adult women. The present study adds to studies that point to the relevance of increasing adaptive emotional regulation in EDs (13), the importance of studying the role of emotions (48), and the psychological factors and processes involved in body dissatisfaction with the aim of improving treatment efficacy (6, 30). Taken together, the present study provides relevant and novel results for the design of prevention and/or treatment programs aimed at increasing body satisfaction among young adult women by helping this population to decrease the use of emotional regulation strategies based on lack of impulse control and the externalization and emotional acceptance of anger. In this way, body dissatisfaction can be prevented from leading to the development of ED.

## Data availability statement

The original contributions presented in this study are included in the article/supplementary material, further inquiries can be directed to the corresponding authors.

## Ethics statement

The studies involving human participants were reviewed and approved by the Deontological Commission of the Faculty of Psychology of the Complutense University of Madrid. The patients/participants provided their written informed consent to participate in this study.

## Author contributions

JM conceived of the presented idea and drafted the manuscript. MH contributed to the analysis and interpretation of data for the manuscript. MG and AE revised the manuscript critically and approved the version to be published. LO and II collected the sample and database. All authors contributed to the article and approved the submitted version.

## Footnotes

1
surveymonkey.com
